# A Reproducible 3D Classification of Orbital Morphology Derived from CBCT and FBCT Segmentation

**DOI:** 10.3390/jcm14217836

**Published:** 2025-11-04

**Authors:** Natalia Bielecka-Kowalska, Bartosz Bielecki-Kowalski, Marcin Kozakiewicz

**Affiliations:** 1 Department of Oral Mucosal and Periodontal Diseases, Medical University of Lodz, 251 Pomorska St., 92-213 Lodz, Poland; natalia.bielecka-kowalska@umed.lodz.pl; 2 Department of Maxillofacial Surgery, Medical University of Lodz, 251 Pomorska St., 92-213 Lodz, Poland; marcin.kozakiewicz@umed.lodz.pl

**Keywords:** orbital morphology, orbit classification, 3D imaging, morphometry

## Abstract

**Background**: Accurate reconstruction of the orbit after trauma or oncological resection requires reliable anatomical references. In unilateral cases, the contralateral orbit can guide repair, but bilateral injuries or pathologies remove this option. To address this problem, we developed a new morphological classification of orbits based on three linear dimensions. **Methods**: A total of 499 orbits from patients of Caucasian descent (age 8–88 years) were analyzed using three-dimensional models generated from cone-beam and fan-beam CT scans. Orbital depth (D), height (H), and width (W) were measured, and proportional indices were calculated. K-means clustering (k = 3) identified recurring morphotypes, validated by linear discriminant analysis (LDA) and supported by ANOVA, Kruskal–Wallis, and correlation tests (age and sex). **Results**: Three morphotypes were identified: Tall & Broad (type A, 33.5%), Deep & Broad (type B, 30.2%), and Compact (type C, 36.2%). All dimensions differed significantly between groups (ANOVA, *p* < 1 × 10^−16^; η^2^ = 0.40–0.51). Male orbits were significantly deeper and wider than female ones (*p* < 0.001). LDA demonstrated excellent separation with 97.5% accuracy. A simplified decision algorithm achieved 82.1% classification accuracy. In situations where only orbital depth could be measured, an alternative cut-off-based method reached 61.5% accuracy, with type B and C better distinguished than type A. **Conclusions**: The proposed classification provides a reproducible framework for describing orbital morphology. It may serve as a reference in cases where local anatomy is disrupted or the contralateral orbit is unavailable. Even millimeter-scale differences in orbital dimensions may correspond to clinically relevant changes in orbital volume and globe position, underlining the potential usefulness of this system in surgical planning.

## 1. Introduction

Reconstruction of the orbit is one of the most difficult tasks in maxillofacial and craniofacial surgery. Trauma and oncologic resections can change the shape of the orbit and cause both esthetic and functional problems. The most common problem is change in globe position, with enophthalmos or exophthalmos. In unilateral cases, surgeons usually use the healthy orbit as a reference for planning. This method is supported by evidence that the orbits are almost symmetrical. In a recent large-scale CT study, Sigron et al. (2024) demonstrated excellent agreement between the right and left orbits in terms of volume, length, and aperture area, confirming the validity of mirror-image planning as a reliable approach in orbital reconstruction [1].

The situation is different in bilateral cases, where both orbits are damaged or deformed. Bilateral orbital fractures are not rare. In the literature, they are reported in 2–6% of smaller series, 17–22% in trauma centers, and up to 30% when complex multi-wall fractures are included [2,3,4]. Similar problems appear in oncology, where tumors of the midface and paranasal sinuses can destroy the orbital walls on both sides [5,6]. Another example is craniofacial fibrous dysplasia, which often involves both orbits, changes bone structure and orbital volume, and makes planning difficult [7]. In all these conditions, the healthy orbit cannot be used as a reference. Despite the growing precision of image-guided and computer-assisted reconstruction, there remains a lack of standardized morphometric references describing orbital proportions. Establishing reproducible normative values for orbital dimensions may improve preoperative assessment, facilitate the design of patient-specific implants, and enhance comparability between studies.

Because of this, there is a need for a simple and reproducible classification of orbital morphology. Such a classification can give reference values for depth, height, and width of the orbit. It can help in surgical planning when both orbits are involved and improve communication between surgeons, radiologists and engineers who design patient-specific implants. This framework may also serve as a reference tool in computer-assisted surgery and implant design, allowing for faster preoperative assessment of orbital proportions. Beyond descriptive anatomy, it provides measurable standards that can support surgical planning when local anatomy is disrupted or the contralateral orbit is unavailable. In this study we present a new three-dimensional classification of the human orbit—Tall & Broad (type A), Deep & Broad (type B), and Compact (type C)—derived from CBCT and FBCT segmentation. Our aim is to provide a tool that is easy to use in clinical practice and useful for further research.

## 2. Materials and Methods

This study was approved by the local bioethics committee (NN/266/11/KB, RNN/267/11/KB, RNN/141/12/KB). The material included 499 orbits from Caucasian patients, aged 8–88 years. Each CT scan provided two orbits, except in cases where image quality or segmentation errors affected one side. Imaging was performed with two scanners: a cone-beam CT (Carestream CS 9300 3D, Carestream Dental LLC, Atlanta, GA, USA) and a fan-beam CT (Aquilion ONE, Toshiba, Otawara, Japan). In total, 66 CBCT and 184 FBCT scans were collected. All data were anonymized before analysis [8].

Cases were taken from the archive of the Maxillofacial Surgery Clinic. Exclusion criteria were: congenital orbital deformities (for example sphenoid wing dysplasia, Goldenhar syndrome), pathological bone changes (e.g., tumors), history of orbital trauma or surgery (including reconstructions and implants), and partial orbital resections. Poor quality scans and studies with strong artifacts were also excluded. Twelve scans were removed because of segmentation errors, which left 499 orbits for the final analysis.

**Figure 1:**
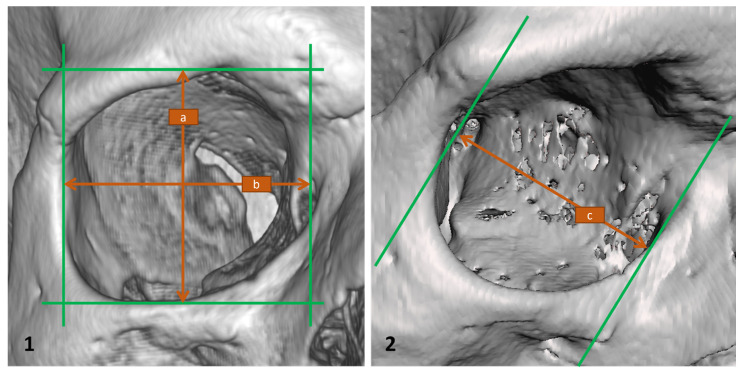
Three-dimensional orbital models used for morphometric analysis. (**1**) Model created in RadiAnt 2024.2 software, frontal projection. Shown measurements: a—orbital height, b—orbital width. (**2**) Model visualized in Meshmixer v.3.5.474, oblique lateral (¾) projection. Shown measurement: c—orbital depth.

Three-dimensional models of the orbit were made from DICOM data by segmentation. For each dataset, global thresholding was adjusted after checking the histogram, according to the method of Baillard and Barillot [9]. The segmented models were used for measurements. All steps were performed with RadiAnt DICOM Viewer (Medixant, Poznań, Poland; www.radiantviewer.com, accessed on 9 April 2024, ver. 2024.1) and Meshmixer (Autodesk, San Rafael, CA, USA; www.autodesk.com, accessed on 9 April 2024, ver. 3.5.474) (Figure 1).

Before measurement, all models were oriented to the Frankfurt horizontal plane and placed in 0-degree frontal view. Orbital walls were marked as perpendicular planes. Linear distances (a, b) were taken in RadiAnt. In Meshmixer the models were rotating to a ¾ lateral projection.

For orbital width, the lateral reference point was set at the most anterior contour of the lateral rim, and the medial reference point corresponded to the anterior lacrimal crest. This approach ensured reproducibility across CBCT and FBCT datasets, as the posterior lacrimal crest is often obscured by bone overlap or scan orientation. All measurements were taken on standardized planes aligned to the Frankfurt horizontal and performed by the same investigator using a consistent procedure to minimize observer-related error.

Orbital height was measured between the superior and inferior rims in the frontal projection. Although this does not represent the maximal vertical dimension deeper in the orbit, the rim-based approach was chosen for its clinical relevance and reproducibility in trauma cases, where internal contours are frequently disrupted. It also corresponds to the region most often reconstructed during orbital wall repair.

Based on the three linear dimensions—depth (D), height (H), and width (W)—three proportional indices were defined to standardize orbital morphology:

- Depth Index (DI) = D/W
- Width Index (WI) = W/H
- Height Index (HI) = H/W

These indices were expressed as dimensionless ratios to allow comparison between imaging modalities (CBCT and FBCT) and between individuals. The same terminology and abbreviations are used consistently throughout the manuscript and figures.

Statistical analysis was carried out using Statgraphics Centurion XVIII. The chi-squared (χ^2^) test was used for categorical data. For continuous data, normality and variance were checked. If assumptions were met, one-way ANOVA was applied, followed by Tukey HSD post hoc tests. If assumptions were not met, the Kruskal–Wallis test was used. To explore potential age-related effects on orbital morphology, the sample was divided into three age groups (8–30, 31–55, and 56–88 years). Differences in orbital height, width, and depth between these groups were tested using the Kruskal–Wallis test with Bonferroni-corrected pairwise comparisons. Correlations with age were assessed using Spearman’s rho. Differences between male and female orbits were assessed using the Mann–Whitney U test, with effect sizes expressed as rank-biserial correlation (r_(rb)_). Sex-related differences in orbital dimensions were analyzed using the Mann–Whitney U test, with effect sizes expressed as rank-biserial correlation (r_(rb)_).

To build the new orbital classification, we used linear discriminant analysis (LDA) based on depth (D), height (H), and width (W). Cut-off values for each type were determined from the discriminant functions and distribution of measurements. The accuracy of classification was tested by reassigning each case and comparing predicted type with the observed one. Effect size was calculated with Cohen’s d for pairwise comparisons. Statistical significance was set at *p* < 0.05.

## 3. Results

A total of 499 orbits from Caucasian patients were analyzed. The study group included 317 males (63.3%) and 182 females (36.7%).

The mean age was 44.6 years (SD ± 18.1), with a median of 40 years. The youngest patient was 8 years old, and the oldest was 88 years old (Table 1).

Three linear dimensions of the orbit—depth (D), height (H), and width (W)—were analyzed. From these parameters, three proportional indices (Depth Index, Width Index, and Height Index, defined in the Materials and Methods section) were calculated.

**Figure 2:**
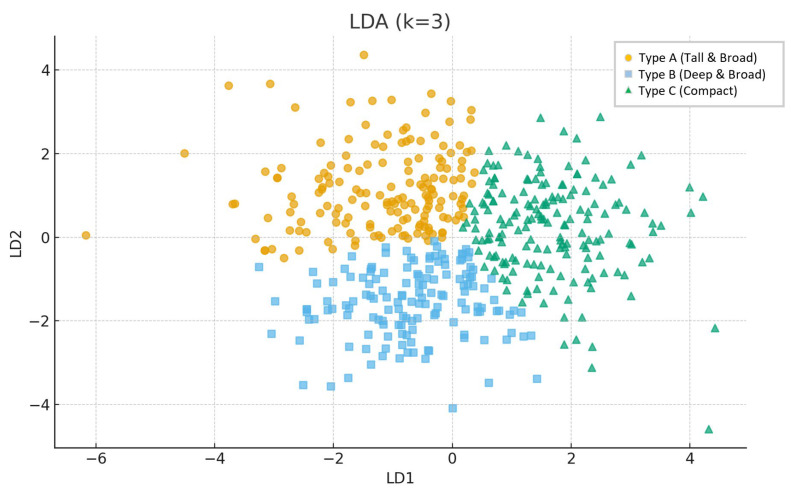
Three-dimensional scatter plot showing distribution of orbital measurements (width, height, depth). The three identified clusters correspond to morphotypes Type A (Tall & Broad), Type B (Deep & Broad), and Type C (Compact).

**Figure 3:**
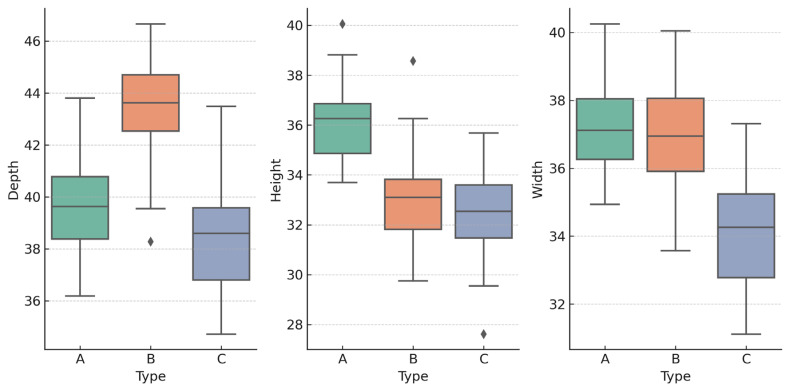
Boxplots of orbital depth (D), height (H), and width (W) by orbital type (A: Tall & Broad, B: Deep & Broad, C: Compact).

After standardization, k-means clustering (k = 3) revealed three reproducible orbital morphotypes: Tall & Broad (type A), Deep & Broad (type B), and Compact (type C). Cluster separation was confirmed with linear discriminant analysis (LDA). Use of LDA to visualize and quantify group separation in craniofacial datasets is common in recent literature [10]. Differences between groups in D, H, and W were highly significant (ANOVA, *p* < 10^−55^) (Figure 2 and Figure 3).

To simplify the use of this classification, a decision tree was applied to raw values. Height (H) was the strongest discriminator, followed by depth (D), with width (W) used as a supporting parameter in borderline cases. The following cut-offs were established:

- If H > 34.35 mm → type A.
- If H ≤ 34.35 mm and D > 41.65 mm → type B.
- If H ≤ 34.35 mm and D ≤ 41.65 mm → type C.

In borderline cases, width supported the classification (W > 34.45 mm = type B, W ≤ 36.25 mm = type C).

The classification accuracy reached 82.1%. Precision and recall were as follows:

- Type A: precision 0.81, recall 0.89
- Type B: precision 0.90, recall 0.69
- Type C: precision 0.78, recall 0.86

Misclassification was most frequent for type B, reflecting partial overlap with the other groups.

The three orbital types differed consistently across all dimensions and proportional indices. Type A was characterized by the greatest orbital height, Type B by the largest depth, and Type C by overall smaller, more compact dimensions. These tendencies were also visible in the index values, which confirmed the geometric distinctness of the groups. (Table 2).

In the studied cohort, the orbital types were distributed as: type A—163 cases (33.5%), type B—147 cases (30.2%), and type C—176 cases (36.2%) (Table 3).

One-way ANOVA confirmed strong differences between the three types:

- D: F(2,483) = 218.18, *p* < 1 × 10^−16^, η^2^ = 0.475
- H: F(2,483) = 256.02, *p* < 1 × 10^−16^, η^2^ = 0.515
- W: F(2,483) = 163.82, *p* < 1 × 10^−16^, η^2^ = 0.404

Kruskal–Wallis tests provided consistent results (all *p* < 1 × 10^−50^). Post hoc Tukey HSD confirmed significant pairwise differences. LDA with 5-fold cross-validation reached 97.5 ± 1.5% accuracy, demonstrating clear separation of the three morphotypes in multidimensional space (Table 4).

When stratified by age, orbital width differed significantly between groups (Kruskal–Wallis H = 11.17, *p* = 0.0038), being narrower in the oldest group (56–88 years) compared with both 8–30 (*p* = 0.007) and 31–55 years (*p* = 0.016). Height and depth showed no significant differences across age groups (*p* = 0.81 and *p* = 0.45, respectively). Weak negative correlations were observed between age and both width (ρ ≈ −0.11, *p* ≈ 0.021) and depth (ρ ≈ −0.11, *p* ≈ 0.025).

**Figure 4:**
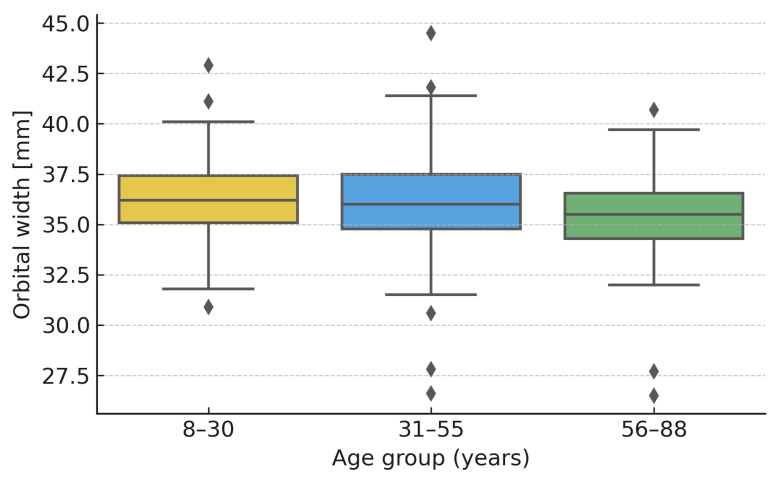
Boxplots showing orbital width (W) across three age groups (8–30, 31–55, 56–88 years). A significant decrease in width was observed in the oldest group (Kruskal–Wallis H = 11.17, *p* = 0.0038).

**Figure 5:**
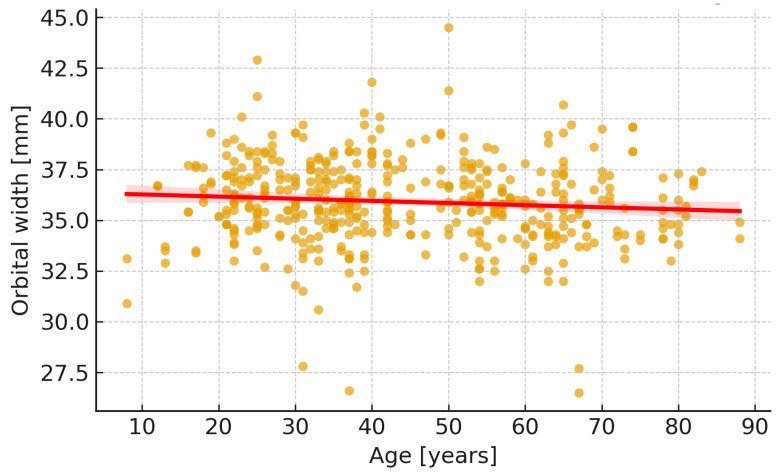
Scatter plot showing correlation between orbital width (W) and age. A weak negative relationship was observed (Spearman’s ρ ≈ −0.11, *p* ≈ 0.021).

These results are illustrated in Figure 4 (orbital width by age group) and Figure 5 (width vs. age correlation). 

**Figure 6:**
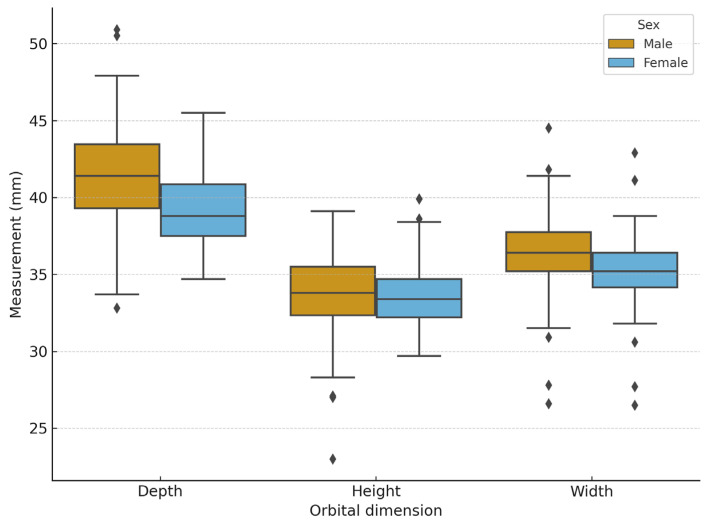
Boxplots showing sex-related differences in orbital dimensions (depth, height, width). Male orbits were significantly deeper and wider than female ones (Mann–Whitney U test, *p* < 0.001 for both).

Male orbits were significantly deeper and wider than female ones (*p* < 0.001 for both), while height showed no significant difference between sexes (*p* = 0.17). Effect sizes indicated moderate sex-related differences in depth (r_(rb)_ = 0.41) and width (r_(rb)_ = 0.38). Figure 6 illustrates these differences.

**Figure 7:**
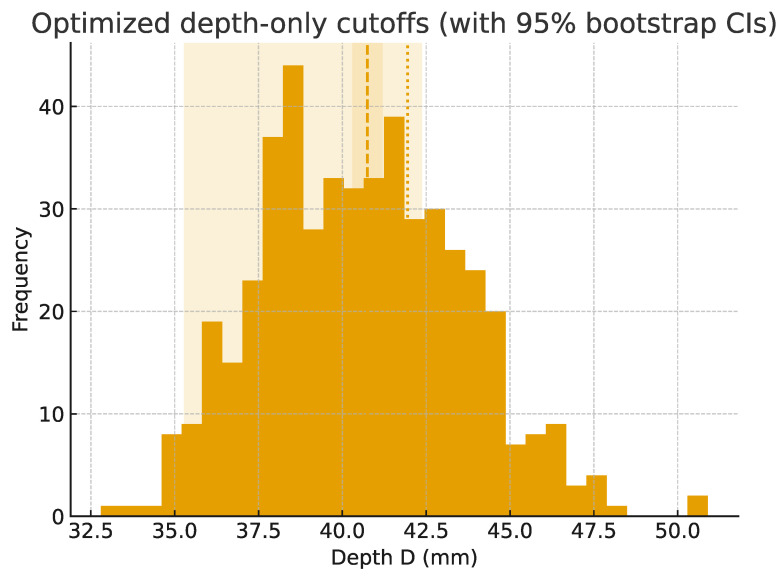
Histogram of orbital depth (D) with optimized cut-off points (D1 = 40.75 mm, D2 = 41.95 mm). The dashed line indicates the mean optimized cut-off value, and the shaded areas represent the 95% bootstrap confidence intervals for the cut-offs.

Because height and width are often unavailable after orbital trauma, an alternative depth-based classification was tested. Two cut-off points were identified: D1 = 40.75 mm and D2 = 41.95 mm. Classification accuracy relative to the full A/B/C system was 61.5% (95% CI: 58.0–64.7%). Precision and recall showed that types B and C were identified more reliably, whereas type A was less distinct when based on depth alone (Figure 7).

## 4. Discussion

In this study, we proposed a new morphological classification of orbits into three reproducible types: Tall & Broad (type A), Deep & Broad (type B), and Compact (type C). The classification is based on objective morphometric measurements, supported by cluster analysis and validated by discriminant analysis. Our approach, where unsupervised clustering was followed by supervised validation and the development of decision rules, reflects strategies already applied in craniofacial morphometrics, where cluster analysis has been used to derive clinically interpretable diagnostic templates [11]. Each type demonstrated statistically significant differences in depth, height, and width, as well as in derived proportional indices. The distribution of the types was balanced, with each occurring in roughly one third of the studied population.

The main clinical value of this classification lies in its potential application in reconstructive and oncological surgery. Orbital fractures, particularly in the inferior and lateral walls, often make it impossible to rely on local anatomy for reconstruction. Previous studies have shown that orbital symmetry is preserved within 0.75 mm in the majority of patients, making the contralateral orbit a natural reference for repair [1]. However, bilateral fractures or tumors involving both orbits remove this point of reference. In such cases, using population-based morphological patterns may provide a useful guideline.

The clinical relevance of millimeter-scale differences should not be underestimated. Orbital volume changes of only 1 cm^3^ can result in approximately 1 mm of enophthalmos or exophthalmos, which is clinically visible and often esthetically disturbing [12]. Our results demonstrate that the three orbital types differ by up to several millimeters in their linear dimensions, which may translate into clinically meaningful differences in orbital volume and globe position [13,14].

This concept may also extend beyond trauma. Tumors of the midface and craniofacial fibrous dysplasia can cause extensive remodeling of orbital walls, making native anatomy unusable for reconstruction [5,6,7]. In such situations, referring to average morphometrics of the corresponding orbital type could improve the planning of resection margins and prosthetic reconstruction.

**Figure 8:**
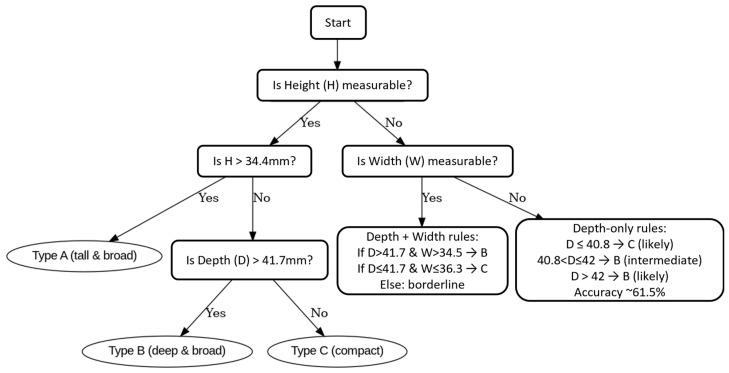
Decision algorithm for orbital classification. Height (H) is the primary determinant, followed by depth (D) and width (W). A simplified depth-only pathway is available when height and width cannot be reliably measured, achieving 61.5% accuracy.

To support clinical use, we developed a stepwise decision algorithm that guides the classification of orbits based on available measurements. The algorithm prioritizes height (H) as the most reliable determinant, followed by depth (D) and width (W). In cases where only depth can be measured, such as after severe orbital trauma, a simplified depth-only pathway is provided. This tool may help surgeons apply the classification consistently, even in incomplete or damaged orbits (Figure 8).

In practical terms, this classification can support orbital surgeons in several ways. It allows estimation of the expected orbital proportions and volume when native anatomy is destroyed, which may guide implant contouring and depth control during reconstruction. In computer-assisted planning, identifying the morphotype can shorten preoperative assessment by providing reference ranges for depth, height, and width. The classification is not intended to replace standard mirror-image planning but to complement it in bilateral or severely comminuted fractures where no healthy reference is available.

**Figure 9:**
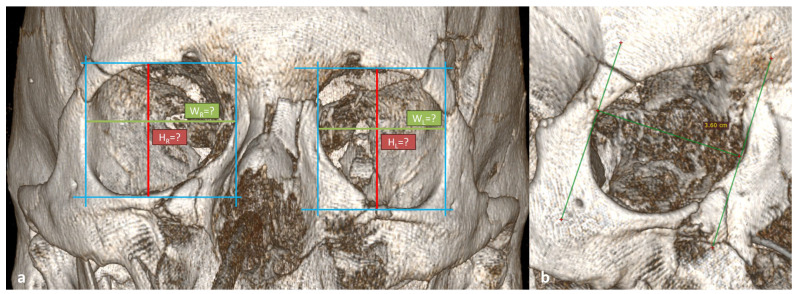
Post-traumatic deformation of both orbits shown on 3D CT reconstruction. (**a**) Frontal projection illustrating the inability to obtain orbital width (W_R_, W_L_) and height (H_R_, H_L_) due to post-traumatic distortion. (**b**) ¾ lateral projection showing the measurement of orbital depth used to assign the orbits to the Compact type according to the proposed classification. Measurements obtained between the auxiliary lines described in the article.

To illustrate the potential clinical application of the proposed classification, we present an example of a post-traumatic case in which both orbits were deformed, preventing reliable measurement of height and width. According to the proposed algorithm, orbital depth was measured from the lacrimal sac impression to the optic canal in a ¾ lateral projection. Based on the obtained depth values, both orbits were classified as Compact type. Knowing the average height and width values for this morphotype allows the surgeon to estimate expected orbital proportions and adjust the reduction in bone fragments accordingly (Figure 9).

**Figure 10:**
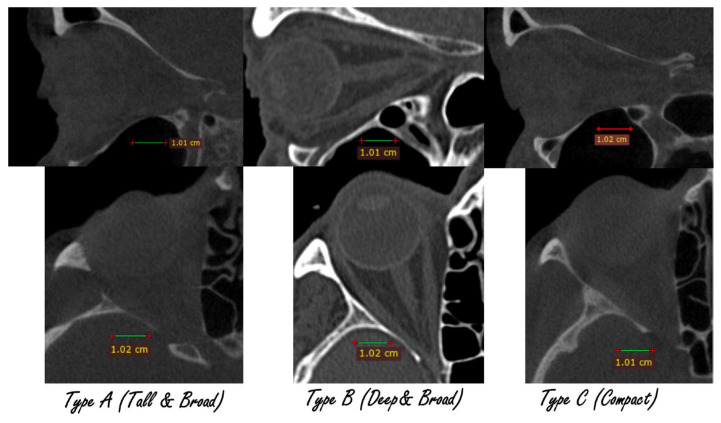
Axial and Sagittal CT cross-sections illustrating typical appearance of the three orbital morphotypes. Differences in orbital height, depth, and width are visible in relation to adjacent bony landmarks.

**Figure 11:**
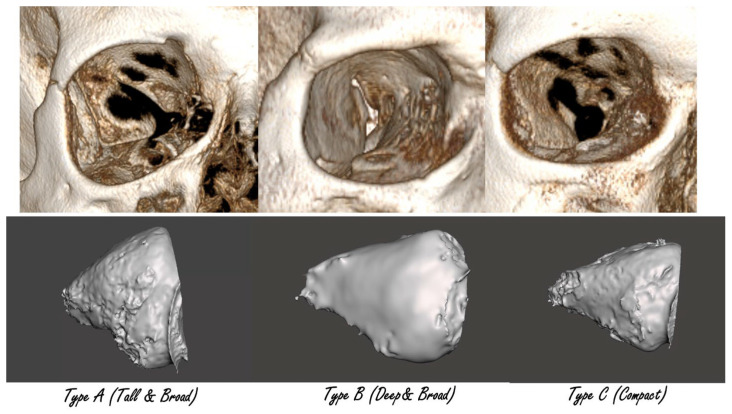
Three-dimensional reconstructions of bone and segmented orbital contents for each morphotype. Volumetric differences and variations in orbital contour are clearly demonstrated.

Our findings are consistent with other reports highlighting the importance of detailed orbital morphometry in surgical planning. Studies on the anterior and posterior ethmoidal arteries and the optic nerve have underlined how anatomical variability can directly affect surgical safety in both orbital and endoscopic skull base surgery [15,16,17]. We selected linear discriminant analysis (LDA) over more complex models because it provides a transparent and interpretable separation of groups, which is important in surgical planning. This choice is consistent with its established role in craniofacial morphometric studies [10,18]. Figure 6 and Figure 7 illustrate how the three orbital types differ in clinical imaging. Figure 6 shows representative CT cross-sections for each type, while Figure 7 presents 3D bone reconstructions with segmented orbital contents. These visualizations underline the practical differences between morphotypes and support the translation of numerical classification into clinical practice (Figure 10 and Figure 11).

A limitation of our work is that the classification is based on linear parameters, which simplify the complex three-dimensional geometry of the orbit. Although the discriminant analysis demonstrated excellent separation (97.5% accuracy), some overlap between types remains, especially for type B. Moreover, the simplified depth-only approach, while clinically useful in trauma cases, reduced accuracy to 61.5%. This trade-off reflects the practical challenge of balancing clinical feasibility with precision.

Despite these limitations, we believe that this classification provides a practical framework for understanding orbital diversity. It may serve as a reference in cases where local or contralateral anatomy is unavailable, and it emphasizes that even subtle morphometric differences can have functional and esthetic consequences.

Although the present study focused on morphometric classification rather than clinical outcomes, the proposed framework is directly applicable to surgical planning and postoperative evaluation. Future research by our group will assess how preoperative morphotype identification correlates with reconstruction accuracy and functional outcomes such as globe position restoration. Another limitation of this study is that all analyzed orbits came from a Caucasian population. Since craniofacial dimensions vary across ethnic groups, the present results should be interpreted with caution when applied to other populations. Further research including non-Caucasian cohorts is needed to verify the generalizability of the proposed classification.

Age-related effects on orbital morphology were modest in this cohort. The slight decrease in orbital width with age, consistent with previous observations of periocular bone resorption, suggests that classification parameters may vary subtly across decades. However, the overall shape proportions (depth–height–width ratios) remained stable, supporting the robustness of the proposed morphotypes across the adult lifespan. In addition to age-related effects, significant sex dimorphism was observed. Male orbits were deeper and wider, consistent with general craniofacial sexual dimorphism reported in previous morphometric studies. These findings emphasize the need to consider sex when applying normative orbital dimensions in surgical planning and implant design.

## 5. Conclusions

This study introduces a new morphological classification of orbits into three reproducible types: Tall & Broad, Deep & Broad, and Compact. The classification is simple, supported by strong statistical evidence, and provides a structured reference for cases where native orbital anatomy cannot be used, such as bilateral trauma or tumors involving both orbits. Although it is based on linear parameters, the identified differences are clinically relevant, as even small dimensional changes may alter orbital volume and globe position. A simplified depth-based version of the classification may also be applied in trauma, where height and width are often unavailable, although with reduced accuracy.

### 5.1. Clinical Implications

The proposed classification offers practical value in surgical planning. In unilateral trauma, the contralateral orbit remains the most accurate reference, but in bilateral fractures or resections this framework provides an alternative guide. Identifying the morphotype can help estimate baseline orbital volume and globe position, supporting virtual planning and the design of patient-specific implants. The depth-only algorithm, despite its lower accuracy, may be especially useful in acute trauma when inferior or lateral walls are destroyed, as orbital depth can usually still be measured reliably.

### 5.2. Future Directions

Further studies are needed to validate this classification across larger and more diverse populations, including non-Caucasian cohorts, to confirm its generalizability. Integration of morphotypes into automated segmentation and surgical navigation systems could enhance intraoperative precision. Volumetric analyses may clarify the relationship between morphotype and changes in orbital volume, improving clinical prediction of enophthalmos and exophthalmos. Finally, the classification can also serve as a basis for creating three standardized orbital models representing the main morphotypes (Tall & Broad, Deep & Broad, and Compact). Such models could be used for pre-bending titanium meshes and designing implants more objectively than the traditional “small–medium–large” approach.

## Figures and Tables

**Table 1:** Demographic characteristics of the study group.

Total Orbits	Sex—Male	Sex—Female	Age, Mean ± SD (years)	Age, Median (years)	Age, Range (years)	Age, 25th Percentile	Age, 75th Percentile
499	317 (63.3%)	182 (36.7%)	44.6 ± 18.1	40	8–88	30	60

SD—standard deviation.

**Table 2:** Descriptive statistics of orbital types (A: Tall & Broad, B: Deep & Broad, C: Compact).

Variable	Type A (Tall & Broad)	Type B (Deep & Broad)	Type C (Compact)
Depth (D) mean ± SD [min–max]	40.1 ± 2.2 [35.3–45.4]	43.52 ± 2.12 [38.5–50.9]	38.56 ± 2.15 [32.8–45.5]
Height (H) mean ± SD [min–max]	35.97 ± 1.45 [32.8–39.9]	32.79 ± 1.52 [27.1–37.1]	32.48 ± 1.66 [23.0–35.8]
Width (W) mean ± SD [min–max]	37.11 ± 1.71 [32.7–44.5]	36.76 ± 1.43 [33.7–39.7]	34.22 ± 1.64 [26.5–37.2]
Depth Index (DI) mean ± SD [min–max]	1.08 ± 0.07 [0.90–1.27]	1.19 ± 0.08 [1.01–1.48]	1.13 ± 0.10 [0.96–1.72]
Width Index (WI) mean ± SD [min–max]	1.03 ± 0.06 [0.84–1.18]	1.12 ± 0.07 [0.98–1.40]	1.06 ± 0.08 [0.77–1.54]
Height Index (HI) mean ± SD [min–max]	0.97 ± 0.06 [0.85–1.19]	0.89 ± 0.06 [0.72–1.02]	0.95 ± 0.07 [0.65–1.29]

**Table 3:** Distribution of orbital types in the study group.

Type	n	%
Type A (Tall & Broad)	163	33.5%
Type B (Deep & Broad)	147	30.2%
Type C (Compact)	176	36.2%

**Table 4:** ANOVA and Kruskal–Wallis results for orbital dimensions.

Variable	ANOVA F(df)	ANOVA *p*	η^2^	Kruskal–Wallis H	KW *p*
Depth (D)	F(2,483) = 218.18	<1 × 10^−16^	0.475	233.27	2.21 × 10^−51^
Height (H)	F(2,483) = 256.02	<1 × 10^−16^	0.515	268.19	5.80 × 10^−59^
Width (W)	F(2,483) = 163.82	<1 × 10^−16^	0.404	227.63	3.72 × 10^−50^

## Data Availability

The original contributions presented in the study are included in the article, further inquiries can be directed to the corresponding authors.
